# Nipah virus and the lessons of COVID-19: are we prepared for the next pandemic threat?

**DOI:** 10.1016/j.imj.2026.100269

**Published:** 2026-06-25

**Authors:** Sharmake Gaiye Bashir, Ilyas Abdullahi khalif, Yusuf Abdullahi Hubow, Ahmed Mohamed Omar, Narura Omar Mohamed, Ayan Abdullahi Mohammed, Hibo hassan Mohamed, Nour Ahmed Dahir, Aniso Mohamed Abdi, Abas Nor Abdi, Yakub Burhan Abdullahi, Mohamed Sharif Abdi, Naima Ibrahim Ahmed, Yusuf Hared Abdi, Ahmed Abdinasir Abdulle

**Affiliations:** a Faculty of Health Science, Salaam University, Mogadishu, Somalia; b Faculty of Health Sciences and Tropical Medicine, Somali National University, Mogadishu, Somalia; c Center for Health Research and Innovation, Somali National University, Mogadishu, Somalia

**Keywords:** Nipah virus, Pandemic preparedness, One health, Zoonotic spillover, Global health governance

## Abstract

The coronavirus disease 2019 (COVID-19) pandemic has exposed profound and persistent weaknesses in global preparedness, including fragmented surveillance systems, governance failures, inequitable access to countermeasures, and erosion of public trust. Although SARS-CoV-2 has dominated global attention, other high-consequence zoonotic pathogens with pandemic potential have remained comparatively neglected. Among these, the Nipah virus (NiV) represents a particularly serious threat, characterized by high case-fatality rates, zoonotic spillover from wildlife reservoirs, documented human-to-human transmission, and the absence of licensed vaccines or specific antiviral therapies. This narrative review synthesizes and contextualizes existing evidence on the NiV through the lens of lessons learned during the COVID-19 pandemic. Drawing on epidemiological data, outbreak experience, and global health policy literature, this review examines the key dimensions of preparedness, including surveillance and diagnostics, laboratory and health-system capacity, vaccine and therapeutic development, emergency governance, ethical decision-making, and the operationalization of One Health approaches, with particular attention to low- and middle-income and fragile settings. The review highlights that despite scientific advances achieved during COVID-19, preparedness for the NiV remains limited and uneven. Persistent gaps in integrated surveillance at human–animal–environment interfaces, constrained laboratory capacity, insufficient investment in countermeasures, fragmented governance, and enduring global inequities threaten timely detection and containment. The NiV should therefore be understood not as an isolated regional concern, but as a warning signal for future “Disease X” scenarios, underscoring the urgent need to translate COVID-19 lessons into sustained, equitable, and multisectoral pandemic preparedness.

## Highlights

• Nipah virus represents a high-fatality zoonotic pathogen with credible pandemic potential and limited global preparedness.
• Lessons from COVID-19 reveal persistent gaps in surveillance, diagnostics, governance, and equitable access to countermeasures.
• Integrated One Health surveillance at human–animal–environment interfaces remains weak and under-operationalized.
• Absence of licensed vaccines or specific antivirals leaves health systems vulnerable to rapid Nipah virus escalation.
• Translating COVID-19 innovations into sustained, equitable preparedness is critical to prevent future “Disease X” scenarios.

## Graphical abstract

Nipah virus combines high fatality, zoonotic spillover, and documented human-to-human transmission, yet remains severely under-prepared for compared with COVID-19. Lessons from COVID-19 on surveillance, health-system capacity, governance, and One Health are translated here into a Nipah-specific preparedness framework, with emphasis on low- and middle-income and fragile settings. Implementing these integrated strategies now is essential to prevent Nipah from becoming the next ‘Disease X’ pandemic threat.

**Figure:**
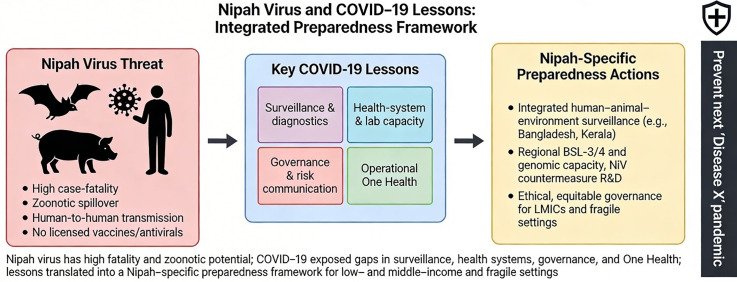

## 1 Introduction

The coronavirus disease 2019 (COVID-19) pandemic has served as an unprecedented global stress test, exposing profound and persistent weaknesses in pandemic preparedness, surveillance, governance, and public trust in health systems worldwide.^1^^,^^2^ Despite decades of warnings about the inevitability of emerging infectious diseases, the rapid spread and devastating impact of SARS-CoV-2 has revealed critical gaps in early detection, coordinated response, and equitable resource allocation, even among nations previously considered well-prepared.^1^^,^^3^ The crisis underscored the limitations of fragmented surveillance systems, underinvestment in public health infrastructure, and consequences of eroded trust between governments and communities.^1^^,^^4^ While COVID-19 has gained global attention, other high-consequence zoonotic pathogens continue to circulate with pandemic potential.^5^, ^6^, ^7^ Among these, Nipah virus (NiV) is a particularly serious threat.^5^^,^^8^ First identified in 1998–1999 during outbreaks in Malaysia and Singapore, NiV is a highly pathogenic paramyxovirus capable of causing severe encephalitis and respiratory illness, with case fatality rates often exceeding 60%.^5^^,^^6^ Its zoonotic nature, transmitted from fruit bats to humans via intermediate hosts or contaminated food, and demonstrated capacity for human-to-human transmission raises concerns about its potential to spark future epidemics or even a global pandemic.^6^^,^^8^ Unlike SARS-CoV-2, however, NiV has not received commensurate global investment or policy attention.^8^^,^^9^ There are no licensed vaccines or specific antiviral therapies for NiV; surveillance remains patchy, and health system readiness for such high-fatality threats is limited, especially in regions where the spillover risk is highest.^6^^,^^8^ Examining the NiV through the lens of the lessons learned from COVID-19 is both timely and essential.^1^^,^^10^ The aim of this narrative review is to critically synthesize and contextualize existing evidence on the NiV as a high-consequence zoonotic threat through the lens of lessons learned from the COVID-19 pandemic. Specifically, this review examines systemic strengths and failures in pandemic preparedness across key domains, including surveillance, diagnostics, health system capacity, governance, risk communication, ethical decision-making, and One Health implementation, with particular attention to low- and middle-income and fragile settings.

## 2 Materials and methods

### 2.1 Study design

This study was conducted as a narrative review aimed at synthesizing and critically interpreting existing evidence on the NiV in the context of lessons learned from the COVID-19 pandemic. A narrative approach was selected to enable integrative analysis across interdisciplinary domains—including epidemiology, surveillance systems, health system preparedness, governance, ethics, and One Health implementation—which are not readily amenable to quantitative synthesis or meta-analysis.

In addition to summarizing existing knowledge, this review adopts an analytical and comparative perspective, explicitly linking lessons from COVID-19 to preparedness gaps for NiV in order to generate policy-relevant insights.

### 2.2 Literature search strategy

A structured literature search was conducted across major scientific databases, including PubMed/MEDLINE, Scopus, Web of Science, and Google Scholar, as shown in Table 1. The search covered publications from January 2018 to January 2026. This timeframe was selected to capture: (1) pre-COVID-19 baseline evidence on NiV and emerging zoonotic threats; (2) pandemic-era literature (2020–2024) that generated key global health lessons, and (3) recent post-pandemic evidence relevant to preparedness and health system resilience.

**Table 1:** Databases and search strategy.

Database	Search strategy (example Boolean query)
PubMed/MEDLINE	(“Nipah virus” OR “Henipavirus”) AND (“COVID-19″ OR “SARS-CoV-2″) AND (“pandemic preparedness” OR “global health security” OR “surveillance” OR “health system resilience” OR “One Health” OR “governance”)
Scopus	TITLE-ABS-KEY (“Nipah virus” OR “Henipavirus”) AND TITLE-ABS-KEY (“COVID-19″ OR “SARS-CoV-2″) AND TITLE-ABS-KEY (“pandemic preparedness” OR “zoonotic spillover” OR “health systems” OR “One Health” OR “ethics” OR “governance”)
Web of Science	TS = (“Nipah virus” OR “Henipavirus”) AND TS = (“COVID-19″ OR “SARS-CoV-2″) AND TS = (“pandemic preparedness” OR “global health” OR “surveillance” OR “One Health” OR “health system resilience”)
Google Scholar	(“Nipah virus” AND “COVID-19 lessons” AND “pandemic preparedness”) OR (“zoonotic spillover” AND “One Health” AND “global health security”)

*Abbreviations*: COVID-19, coronavirus disease 2019; SARS-CoV-2, severe acute respiratory syndrome coronavirus 2.

Search terms were developed iteratively and combined using Boolean operators. Core concepts included: “Nipah virus”, “Henipavirus”, “COVID-19″, “SARS-CoV-2″, “pandemic preparedness”, “zoonotic spillover”, “One Health”, “global health security”, “surveillance”, “health system resilience”, “governance”, and “ethics”. Additional relevant studies were identified through manual screening of reference lists of selected articles and key reports from international health organizations.

### 2.3 Eligibility criteria

Studies were selected based on their relevance to the objectives of the review. Inclusion criteria were: (1) Publications addressing NiV epidemiology, transmission, outbreak response, or prevention strategies; (2) Studies examining COVID-19 preparedness, response, or health system performance; (3) Literature focusing on pandemic preparedness frameworks, One Health approaches, governance, or ethical considerations; (4) Studies relevant to low- and middle-income countries (LMICs) and fragile settings; (5) Peer-reviewed articles, systematic reviews, policy analyses, and high-quality reports published in English between 2018 and 2026. Exclusion criteria included: (1) Studies not directly related to zoonotic diseases or pandemic preparedness; (2) Articles lacking methodological clarity or scientific rigor; (3) Non-scholarly sources without clear institutional credibility.

### 2.4 Study selection and synthesis

The study selection process followed a two-stage screening approach. First, titles and abstracts were screened to identify potentially relevant studies. Second, full-text articles were assessed against the predefined eligibility criteria. Although this review does not follow a formal systematic review protocol, efforts were made to ensure transparency, consistency, and comprehensiveness in study selection. Data were synthesized using a thematic analysis approach, with findings organized into key domains of pandemic preparedness, including: (1) Surveillance and early warning systems; (2) Laboratory and diagnostic capacity; (3) Health system resilience; (4) Governance and policy coordination; (5) Ethical considerations; (6) One Health implementation.

### 2.5 Quality appraisal and evidence interpretation

A formal risk-of-bias assessment was not conducted, consistent with narrative review methodology. However, the quality and relevance of included studies were critically evaluated during the synthesis process. Priority was given to: (1) Peer-reviewed publications in reputable journals; (2) Studies with clearly defined methodologies and robust data; (3) Evidence derived from real-world outbreak settings (e.g., Bangladesh, India); (4) Reports from authoritative global health organizations. Findings were compared across sources to identify consistencies, divergences, and knowledge gaps, with greater emphasis placed on well-substantiated evidence. Less rigorous sources were interpreted cautiously.

### 2.6 Analytical framework

To enhance originality and analytical depth, findings were interpreted using a comparative framework linking COVID-19 lessons to NiV preparedness gaps. This framework enabled: (1) Identification of systemic weaknesses revealed during COVID-19; (2) Mapping of these weaknesses onto current vulnerabilities in NiV preparedness; (3) Development of context-specific and actionable policy recommendations, particularly for LMICs and high-risk regions.

## 3 NiV as an emerging pandemic threat

### 3.1 Virology and natural reservoir

The NiV is one of the most formidable pandemic threats among emerging zoonotic pathogens, combining a high case-fatality rate, broad host range, capacity for human-to-human transmission, and lack of effective medical countermeasures. Its unpredictable spillover from wildlife reservoirs, rapid clinical progression, and potential for mutation underscore the urgent need for global vigilance and preparedness, especially in light of the lessons learned from the COVID-19 pandemic.^7^^,^^11^ Virology and natural reservoir NiV is a highly pathogenic, negative-sense, single-stranded RNA virus belonging to the genus Henipavirus within the *Paramyxoviridae* family.^9^^,^^12^ Its natural reservoir is fruit bats of the genus *Pteropus*, which are widely distributed across Asia and Oceania.^12^^,^^13^ These bats harbor NiV asymptomatically but shed the virus in urine and saliva, and partially eaten fruit, facilitating environmental contamination and zoonotic spillover.^7^^,^^12^ The broad geographic range of *Pteropus* bats means that large swathes in South and Southeast Asia and potentially beyond are at risk for future outbreaks.^13^^,^^14^

### 3.2 Transmission pathways

NiV is primarily transmitted to humans through direct contact with infected bats or their secretions (notably via the consumption of contaminated fruit or raw date palm sap), as well as through intermediate hosts such as pigs or other domestic animals.^7^^,^^12^ Critically, multiple outbreaks have demonstrated efficient human-to-human transmission via close contact with bodily fluids or respiratory secretions, particularly in healthcare settings and among caregivers.^12^^,^^15^ While NiV is not currently considered airborne in the same way as SARS-CoV-2, its ability to spread between people raises significant concerns for amplification in densely populated regions.^12^^,^^15^

### 3.3 Clinical severity and case-fatality rates

NiV infection in humans presents with a spectrum ranging from asymptomatic infection to acute respiratory illness and rapidly progressive encephalitis. The disease often begins with non-specific symptoms, such as fever, headache, myalgia, and sore throat, but can quickly progress to severe neurological complications, including seizures, coma, and death.^12^^,^^16^ Case-fatality rates are alarmingly high: estimates range from 40% to over 90%, depending on the outbreak context and healthcare access.^7^^,^^11^ Survivors may experience long-term neurological sequelae.

### 3.4 Features heightening pandemic risk

As shown in Table 2, several characteristics make NiV particularly dangerous from a pandemic-preparedness perspective.

**Table 2:** Features heightening the pandemic risk of Nipah virus.

Feature	Description	References
High lethality	With fatality rates far exceeding those of most respiratory viruses, including SARS-CoV-2, Nipah virus outbreaks can devastate affected communities.	^7^^,^^11^
Zoonotic versatility	The virus’s ability to infect multiple species (bats, pigs, horses, cats) increases opportunities for cross-species transmission and viral adaptation.	^7^
Human-to-human transmission	Documented chains of person-to-person transmission raise the risk of larger outbreaks if the virus is introduced into urban or healthcare settings.	^7^^,^^17^
Genetic plasticity	As an RNA virus with error-prone replication machinery, Nipah virus has significant potential for mutation. A strain with increased transmissibility but lower lethality could facilitate sustained human transmission, a scenario reminiscent of early SARS-CoV-2 evolution.	^9^^,^^18^
Lack of countermeasures	There are currently no licensed vaccines or specific antiviral therapies for Nipah virus; treatment remains supportive only. This leaves populations vulnerable to rapid escalation should containment fail.	^9^^,^^11^
Diagnostic challenges	Early clinical symptoms often mimic other febrile illnesses, and delayed recognition can significantly hinder timely outbreak detection and response.	^8^

*Abbreviation*: SARS-CoV-2, severe acute respiratory syndrome coronavirus 2.

### 3.5 Contrasts with respiratory viruses like SARS-CoV-2

While both NiV and SARS-CoV-2 are zoonotic RNA viruses capable of human-to-human transmission, key differences shape their pandemic potential, as shown in Table 3.

**Table 3:** Contrasts between Nipah virus and SARS-CoV-2 relevant to pandemic potential.

Dimension	Description	References
Transmission efficiency	SARS-CoV-2 spreads primarily via respiratory droplets and aerosols with high efficiency, whereas Nipah virus person-to-person transmission is less efficient but still significant, particularly in close-contact settings.	^16^
Case-Fatality rate	Nipah virus mortality far exceeds that observed for COVID-19, contributing to its classification as a high-consequence pathogen.	^14^
Outbreak pattern	Nipah virus outbreaks have been sporadic and geographically limited to date; however, increasing encroachment into bat habitats and expanding global travel could rapidly alter this dynamic.	^14^
Preparedness gaps	The world was unprepared for COVID-19 despite decades of coronavirus research. For Nipah virus, a comparatively less-studied pathogen, gaps in surveillance, diagnostics, therapeutics, and public awareness are even more pronounced.	^15^

*Abbreviations*: COVID-19, coronavirus disease 2019; SARS-CoV-2, severe acute respiratory syndrome coronavirus 2.

### 3.6 Epidemiology and outbreak experience

NiV outbreaks have predominantly affected South and Southeast Asia, spanning Bangladesh, India, Malaysia, Singapore, and sporadic cases in the Philippines, with potential niches covering 19% of the region’s land mass driven by *Pteropus* bat reservoirs.^14^ Pre-COVID-19 patterns revealed recurrent winter-seasonal spillovers in Bangladesh linked to raw date palm sap consumption contaminated by bat secretions, alongside foodborne zoonoses in Malaysia via pigs, escalating to human-to-human transmission through respiratory droplets and body fluids, yielding case fatality rates of 40%–75%.^19^^,^^20^ Health system responses are often reactive and hampered by surveillance confined to sentinel hospitals, resulting in under-reporting of nearly half of outbreaks due to geographic remoteness and delayed diagnostics, exposing structural frailties, such as inadequate biosafety and contact tracing in resource-poor settings.^21^ These vulnerabilities manifested in superspreader events, particularly hospital-based clusters where unprotected close contacts fueled amplification, underscoring missed opportunities for health integration and proactive bat surveillance.^22^ Post-COVID-19, enhancements include Bangladesh’s nationwide active surveillance expansion since 2018, incorporating enhanced sub-district monitoring and molecular diagnostics alongside Kerala’s (India) rapid BSL-2/3 deployment, intersectoral coordination, and compassionate broad-spectrum antivirals such as remdesivir, averting larger outbreaks despite ongoing spillovers.^21^^,^^22^ However, persistent gaps recurring under detection in non-sentinel areas, limited NiV-specific countermeasures, and ecological disruptions from deforestation signal incomplete transformation.^23^^,^^24^ COVID-19′s legacy of surged diagnostics and vaccines offers cross-applicable tools, but without sustained investment in NiV-tailored vaccines, monoclonal antibodies, and community interventions, neglectful patterns endure, imperiling pandemic readiness.^22^^,^^25^

## 4 COVID-19 as a global preparedness stress test

The COVID-19 pandemic has served as an unprecedented systemic stress test of global and national preparedness, exposing critical vulnerabilities across early warning systems, political decision-making, health-system surge capacity, workforce resilience, risk communication, misinformation management, and global equity.^26^^,^^27^ Rather than a disease-specific event, COVID-19 revealed the limitations of existing frameworks and underscored the urgent need for adaptable, integrated strategies to confront future zoonotic threats, such as the NiV.^2^^,^^26^

Despite the existence of preparedness indices, such as the Global Health Security Index, the pandemic demonstrated that no country was fully equipped to manage a crisis of this magnitude.^28^^,^^29^ High Global Health Security Index scores did not consistently translate into effective responses; some countries with robust theoretical preparedness struggled with the detection, containment, and equitable access to health products.^28^^,^^30^ This disconnect highlights the inadequacy of static metrics and the necessity for dynamic evaluation tools that incorporate governance quality, political leadership, public trust, and sociocultural factors.^28^^,^^29^

Failures in early warning systems were evident in the delayed detection and reporting of SARS-CoV-2, and ^26^^,^^31^ the rapid adaptation of standardized protocols such as the WHO UNITY studies enabled more coordinated epidemiological investigations but also revealed gaps in surveillance infrastructure and data sharing, particularly in LMICs.^32^ Political decision-making often lags behind scientific evidence. Inconsistent policies on non-pharmaceutical interventions, testing strategies, and vaccine rollouts contribute to preventable morbidity and mortality.^33^^,^^34^ The health system surge capacity was rapidly overwhelmed, even in high-income settings.^27^ Shortages of personal protective equipment, ventilators, ICU beds, and trained personnel have exposed chronic underinvestment in public health infrastructure.^26^

Workforce resilience has emerged as a link pin for an effective response. The pandemic’s protracted course has led to widespread burnout among healthcare workers and highlighted the need for flexible staffing models and psychosocial support systems.^35^^,^^36^ Risk communication faltered amid an “infodemic” of misinformation; public trust in authorities varied widely by country and was further eroded by politicization of health messaging.^2^^,^^26^ Despite scientific advances, vaccine hesitancy persisted, emphasizing the importance of culturally sensitive communication strategies.^28^^,^^34^

Global equity failures were stark; disparities in access to diagnostics, therapeutics, and vaccines prolonged transmission cycles in low-resource settings and increased the risk of variant emergence.^28^^,^^37^ The pandemic underscored that preparedness is not merely a function of resources, but also of governance structures capable of rapid adaptation and inclusive decision-making.^27^^,^^29^ These systemic failures provide important insights for understanding preparedness gaps in NiV.

## 5 Applying COVID-19 lessons to NiV preparedness

The COVID-19 pandemic has exposed critical vulnerabilities in global health systems, many of which remain unaddressed and could have even more catastrophic consequences in the event of a Nipah outbreak. While SARS-CoV-2 preparedness has benefited from rapid scientific mobilization, robust surveillance, and unprecedented vaccine development, current preparedness for the NiV is markedly less advanced across key domains: Surveillance readiness, diagnostic capacity, laboratory networks, vaccine and therapeutic development, emergency governance, and international coordination.

The emergence of SARS-CoV-2 has been met with the rapid scaling of genomic surveillance and diagnostic infrastructure worldwide. Global data-sharing platforms enable real-time tracking of viral evolution and facilitate swift adaptation of public health responses.^38^ In contrast, NiV surveillance remains limited to sporadic outbreak regions, with minimal integration into global networks. Means that the early detection of Nipah spillover events is unlikely.^39^ The diagnostic capacity for SARS-CoV-2 has rapidly expanded through the deployment of high-throughput polymerase chain reaction assays and innovative point-of-care tests,^40^ whereas Nipah diagnostics are constrained by biosafety level 4 (BSL-4) requirements and limited laboratory availability, impeding timely case identification and containment.^9^^,^^41^

Laboratory networks for SARS-CoV-2 were rapidly established or repurposed and supported by international collaborations and funding mechanisms. However, research on the NiV is hampered by its BSL-4 classification and the global scarcity of suitable facilities.^9^^,^^42^ This restricts both basic virological research and the evaluation of candidate therapeutics or vaccines. The COVID-19 experience demonstrated that prior investment in platform technologies (e.g., mRNA vaccines) enabled record-breaking vaccine development timelines.^32^^,^^43^ In contrast, there are currently no licensed vaccines or therapeutics for NiV; most candidates remain in preclinical or early clinical stages due to limited funding, regulatory hurdles, and insufficient translational infrastructure.^9^^,^^41^

Emergency governance during COVID-19 revealed strengths such as rapid mobilization under WHO leadership and profound weaknesses: Fragmented policy responses, inequitable resource allocation, and failures in cross-border coordination exacerbate morbidity and mortality.^38^^,^^44^ These failures would likely be magnified in a NiV scenario, given its high case-fatality rate (40%–80%), potential for nosocomial amplification, and the fragility of health systems in endemic regions.^9^^,^^41^ Potential for nosocomial amplification and the fragility of health systems in endemic regions. The absence of a scalable surge capacity for critical care would likely result in an overwhelming collapse of the health system.

International coordination mechanisms that emerged during COVID-19, such as COVID-19 Vaccines Global Access for vaccine distribution, were essential, but ultimately insufficient, to ensure equitable access. For the NiV, such frameworks are largely theoretical, and there is no global stockpile or distribution plan for diagnostics or countermeasures.^9^^,^^38^ Moreover, the lack of standardized protocols for data sharing or sample exchange impedes coordinated response efforts.

## 6 Conceptual framework for NiV preparedness

To address the limitations identified in existing pandemic preparedness approaches, this review proposes a conceptual framework that systematically links lessons learned from the COVID-19 pandemic to critical gaps in NiV preparedness. This framework is intended to move beyond descriptive synthesis toward a structured, actionable model for strengthening global and regional readiness for high-consequence zoonotic threats.

The framework is grounded in evidence demonstrating that COVID-19 exposed systemic weaknesses across multiple domains, including fragmented surveillance systems, insufficient laboratory capacity, weak governance structures, inequitable access to countermeasures, and failures in risk communication and public trust.^26^, ^27^, ^28^ These weaknesses are not unique to COVID-19 but are highly relevant to NiV preparedness, where similar vulnerabilities persist, particularly in endemic and high-risk regions. The proposed framework organizes preparedness into three interconnected layers.

### 6.1 Systemic lessons from COVID-19

The first layer captures key systemic failures observed during the COVID-19 pandemic, including delayed detection, lack of integrated surveillance, inadequate surge capacity, fragmented governance, and global inequities in access to diagnostics and vaccines.^26^^,^^27^ These lessons provide a critical evidence base for rethinking preparedness beyond disease-specific responses.

### 6.2 Mapping to NiV preparedness gaps

The second layer explicitly maps these COVID-19 lessons onto existing gaps in NiV preparedness. For example, the lack of real-time genomic surveillance observed during COVID-19 corresponds to the absence of integrated human–animal surveillance systems for NiV. Similarly, inequities in vaccine distribution during COVID-19 highlight the risks associated with the absence of licensed vaccines and limited therapeutic pipelines for NiV.^9^^,^^41^ This mapping establishes clear correspondences between past failures and future risks, addressing a key gap identified in current literature.

### 6.3 Actionable preparedness strategies

The third layer translates these mapped insights into actionable and scalable strategies, particularly for LMICs and fragile settings. These include: (1) Strengthening integrated One Health surveillance systems across human–animal–environment interfaces ^45^^,^^46^; (2) Expanding regional laboratory and diagnostic capacity, including BSL-3/4 infrastructure and decentralized testing; (3) Accelerating investment in vaccines and therapeutic platforms for emerging zoonotic pathogens; (4) Enhancing governance, coordination, and accountability mechanisms for outbreak response; (5) Promoting community engagement and risk communication to strengthen public trust and compliance.

**Fig. 1:**
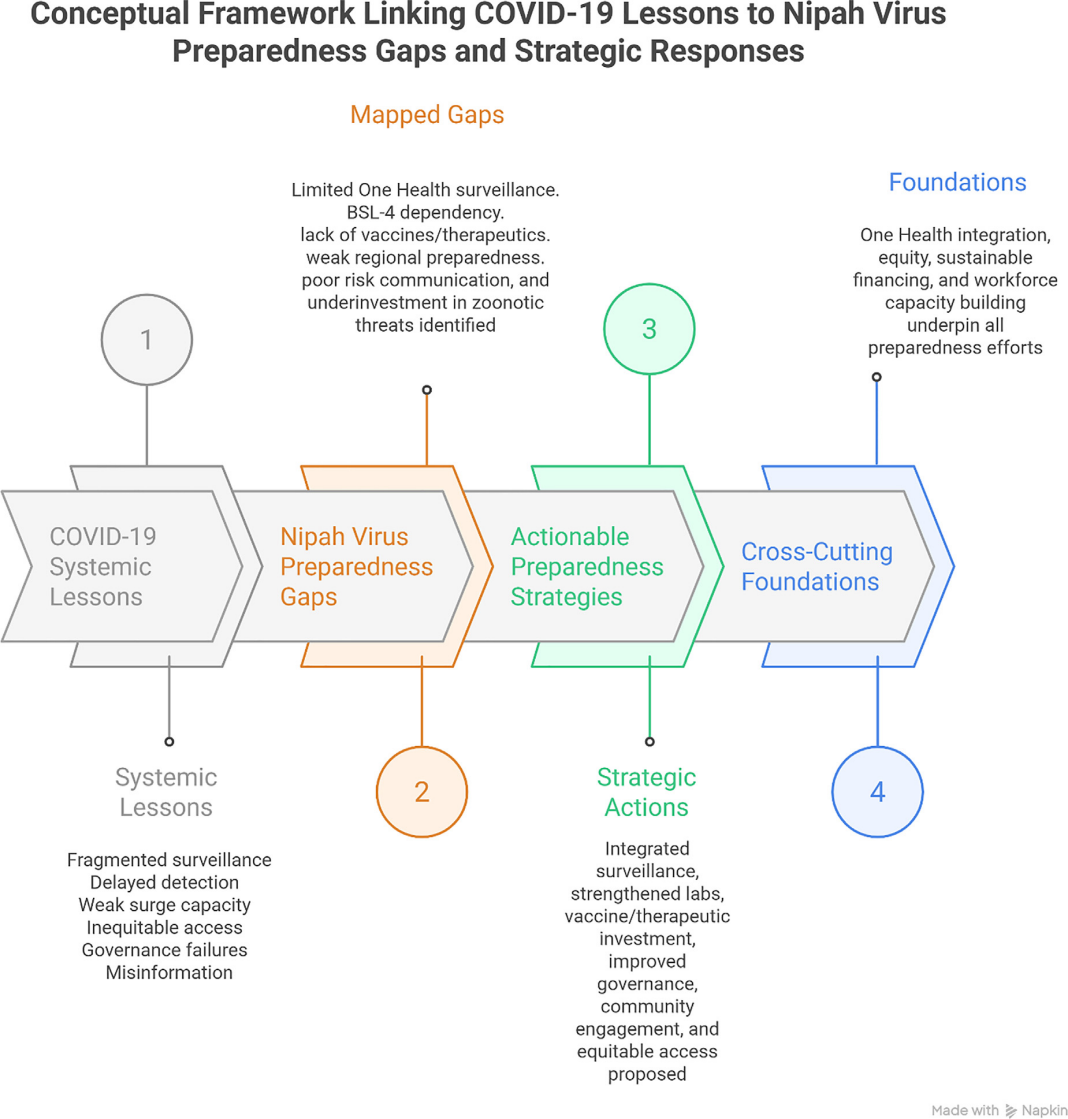
Conceptual framework linking COVID-19 lessons to Nipah virus preparedness gaps and actionable strategies. Abbreviation: BSL-4, biosafety level 4.

Unlike conventional narrative approaches, this framework (Fig. 1) emphasizes the dynamic interaction between past pandemic experiences and future preparedness needs, positioning NiV not as an isolated threat but as a critical test case for global readiness in the era of emerging “Disease X” scenarios. By linking systemic lessons, identified gaps, and targeted interventions, the framework provides a coherent and scalable model for strengthening pandemic preparedness across diverse settings.

## 7 One health in practice: beyond rhetoric

The emergence of the NiV at the human–animal–environment interface, compounded by lessons from the COVID-19 pandemic, underscores the urgent need to move One Health from conceptual endorsement to operational reality.^45^^,^^47^ Despite widespread recognition of its value, One Health issue remains under-implemented globally, with persistent gaps in surveillance, governance, and cross-sectoral coordination that have repeatedly hampered effective responses to zoonotic threats such as the NiV.^48^^,^^49^

NiV spillover events emblematic of the complex interplay between bat ecology, land-use change, agricultural intensification, and urbanization.^14^^,^^50^ Deforestation and habitat fragmentation have increased the contact between fruit bats (*Pteropus spp.*), livestock, and humans, facilitating viral transmission across species boundaries,^50^^,^^51^ which further exacerbates these risks by disrupting ecological balances and creating new interfaces for pathogen emergence.^50^^,^^51^ However, despite clear scientific evidence linking environmental change to zoonotic risk, most One Health initiatives remain siloed within human or animal health sectors, with environmental dimensions often marginalized or neglected.^49^^,^^52^

The COVID-19 pandemic exposed the high cost of fragmented surveillance systems and governance structures.^53^ Siloed data collection and poor interdepartmental communication delayed outbreak detection and response in multiple settings.^48^^,^^53^ The lack of integrated early warning systems capable of synthesizing signals from human, animal, and environmental health domains meant that opportunities for timely intervention were missed.^47^^,^^48^ These failures were not unique to COVID-19; similar patterns have been observed in repeated Nipah outbreaks in South and Southeast Asia, where weak cross-sectoral collaboration impeded coordinated action.^50^^,^^54^

Barriers to operationalizing one’s health are multifaceted.^45^^,^^49^ At the policy level, conflicting departmental priorities and limited institutional capacities hinder sustained collaborations.^48^^,^^49^ Resource constraints, financial, technical, and human, are pervasive in both high- and low-income settings.^48^^,^^49^ Even when frameworks exist on paper, real-time data sharing is hampered by bureaucratic inertia and inconsistent standards,^48^^,^^53^ workforce shortages, and insufficient multidisciplinary training, further undermining implementation efforts.^45^^,^^48^ Critically, community engagement is often superficial or absent; local knowledge systems are rarely integrated into decision-making processes despite their importance in contextualizing interventions.^49^^,^^55^ The proliferation of One Health network since COVID-19 has not translated into equitable investments or balanced stakeholder representation.^45^^,^^52^ Most networks remain concentrated in Europe or North America; emerging infections dominate agendas at the expense of endemic threats that affect marginalized populations elsewhere.^45^^,^^49^ Environmental health professionals continue to be underrepresented in governance structures, which is a legacy of One Health’s historical focus on human and animal health.^45^^,^^52^

## 8 Ethical and governance issues

### 8.1 Ethical foundations of pandemic preparedness

The ethical responsibilities associated with preparedness for high-risk pathogens such as the NiV have been brought into sharp relief by the COVID-19 pandemic, which exposed both the strengths and failures of global and national governance systems.^10^^,^^56^ At the heart of these challenges is the imperative to act decisively in the face of scientific uncertainty, balancing precautionary principles with proportionality, transparency, and accountability.^57^^,^^58^ The ethics of delayed action and political inaction are not merely theoretical concerns; they have tangible consequences for public trust, legitimacy, and health outcomes, particularly in vulnerable populations.^59^^,^^60^

### 8.2 Governance failures and systemic weaknesses

Lessons from COVID-19 underscore that effective pandemic preparedness is inseparable from robust ethical frameworks and governance mechanisms that prioritize equity, inclusiveness, and evidence-based decision making.^56^^,^^61^ Ethical frameworks for pandemic preparedness emphasize a constellation of values: Equity, justice, reciprocity, transparency, stewardship, and solidarity.^56^^,^^62^ These principles guide the allocation of scarce resources and inform decisions regarding when to act under conditions of uncertainty. The precautionary principle of acting to prevent harm, even when full scientific certainty is lacking, has been widely invoked but inconsistently applied.^57^^,^^58^ Delayed or inadequate responses often reflect failures in risk governance, such as insufficient risk communication, a lack of transparent data sharing, weak negotiation between actors, and limited public participation.^10^^,^^63^ Such failures can amplify social inequality and erode trust in institutions.^10^^,^^64^

### 8.3 Political dynamics and accountability

Political decision-making plays a critical role in shaping pandemic responses. Political inaction during pandemics is frequently shaped by competing incentives for credit and blame among the leaders.^60^^,^^65^ Centralization may be used to claim credit for decisive actions; decentralization may serve to deflect responsibility for unpopular outcomes.^60^^,^^65^ This dynamic can undermine coherent responses and foster a “governance by chaos” where confusion prevails over coordinated action.^66^^,^^67^ Moreover, the absence of clear accountability mechanisms, both nationally and globally, has hampered efforts to enforce compliance with ethical standards or international agreements.^57^^,^^68^

### 8.4 Trust, transparency, and global governance

Transparency is fundamental to ethical governance.^56^^,^^62^ Public trust hinges on government willingness to openly communicate about risks, uncertainties, and rationales for policy choices.^10^^,^^60^ When transparency falters or decisions are perceived as opaque or politically motivated rather than evidence-based, legitimacy suffers.^69^^,^^70^ The COVID-19 experience demonstrated that trust is not only a moral good but also a practical necessity; it shapes public compliance with health measures and ultimately determines the effectiveness of response strategies.^64^^,^^71^ Accountability mechanisms remain underdeveloped at both the national and international levels.^57^^,^^72^ The ongoing negotiations around a global pandemic treaty highlight persistent gaps in enforcement provisions; without robust compliance structures, treaties risk becoming “empty promises” that fail to deliver meaningful protection against future threats.^57^^,^^68^ Renewed calls for global health diplomacy emphasize the need for shared responsibility, equity considerations, and enforceable commitments as prerequisites for resilient pandemic governance.^10^^,^^56^

## 9 Implications and future directions for LMICs and fragile settings

The preparedness of LMICs and fragile settings for a potential NiV outbreak is profoundly shaped by persistent limitations in surveillance, laboratory capacity, workforce availability, and overall health system fragility.^73^^,^^74^ The COVID-19 pandemic has starkly illuminated these vulnerabilities, revealing deep-seated inequities that are likely to re-emerge during future outbreaks of high-threat pathogens, such as Nipah.^75^^,^^76^ Surveillance systems in many LMICs remain fragmented, under-resourced, and often lack the integration necessary for timely detection and response to emerging infectious diseases.^73^^,^^77^ Inadequate digital infrastructure, inconsistent data collection, and limited interoperability between surveillance platforms impede the rapid identification of outbreaks.^77^^,^^78^ While digital tools have shown promise in improving data quality and timeliness, their effectiveness is frequently constrained by poor connectivity, insufficient training, and a lack of sustained government support.^78^ The experience of COVID-19 demonstrates that reliance on long-term surveillance alone is insufficient; adaptive strategies that bridge evidence-practice gaps are essential for effective preparedness.^76^^,^^78^

Laboratory capacity constraints further undermine readiness for the outbreak.^73^^,^^77^ Many LMICs face shortages in essential diagnostic equipment, reagents, and skilled personnel. During COVID-19, the global competition for diagnostics disproportionately disadvantaged LMICs, and only a fraction of global diagnostic tests reached these settings despite their large populations.^75^ Innovative approaches, such as repurposing existing diagnostic platforms (e.g., GeneXpert) and introducing point-of-care testing, have been successfully implemented in some fragile contexts such as Somalia, but these remain exceptions rather than the norm.^75^ Sustained investment in laboratory infrastructure, including genomic sequencing capabilities, is critical for enabling real-time surveillance and effective outbreak control.^76^^,^^79^

Workforce shortages represent a chronic barrier to health security.^74^^,^^80^ The flight of human capital due to safety concerns, inadequate remuneration, and difficult working conditions is exacerbated during crises;^73^^,^^78^ task shifting, redeployment of retired staff, and targeted training initiatives have been adopted with varying degrees of success during recent emergencies.^81^^,^^82^ However, high turnover rates and insufficient technical capacity continue to impair the quality of data collection, timeliness of reporting, and overall system resilience.^73^^,^^78^

Health system fragility characterized by weak governance structures, underfunding, poor infrastructure, and limited surge capacity renders LMICs particularly susceptible to being overwhelmed by public health emergencies.^74^^,^^80^ The COVID-19 pandemic exposed how disruptions in routine care disproportionately affected marginalized populations; similar patterns were anticipated when a Nipah outbreak occurred.^81^^,^^83^ Efforts to strengthen health systems must prioritize context-specific interventions that address both acute shocks and chronic vulnerabilities without relying on unsustainable investments or idealized models that are ill-suited to local realities.^76^^,^^84^

Equity is a central concern. The uneven distribution of resource diagnostics, vaccines, therapeutics, and the diversion of attention from non-pandemic health needs have deepened pre-existing social and health inequalities within and between countries.^76^^,^^85^ Pandemic responses that fail to account for these disparities risk perpetuating cycles of vulnerability among marginalized groups.^80^

The persistent threat of NiV requires a shift from reactive to anticipatory and integrated pandemic preparedness. Building on lessons from COVID-19, this review identifies key priorities for strengthening readiness, particularly in high-risk and resource-constrained settings.

First, integrated surveillance systems must be operationalized through One Health approaches that link human, animal, and environmental data streams to enable early detection of spillover events. This includes expanding community-based surveillance and strengthening real-time data-sharing mechanisms across sectors. Second, laboratory and diagnostic capacity must be scaled in endemic regions through investment in BSL-3/4 infrastructure, decentralized testing platforms, and workforce training, ensuring timely detection and response. Third, accelerating countermeasure development is critical. Leveraging COVID-19 advances, investment should prioritize adaptable vaccine platforms (e.g., mRNA and viral vectors), alongside monoclonal antibodies and repurposed antivirals, supported by flexible regulatory pathways for outbreak settings. Fourth, governance and coordination mechanisms must be strengthened through clear accountability frameworks, regional collaboration, and pre-established protocols for data sharing, resource mobilization, and emergency response. Fifth, context-specific implementation in LMICs and fragile settings is essential. Evidence from Bangladesh and India, demonstrates that integrated surveillance, rapid diagnostics, and coordinated response systems can significantly mitigate outbreak impact. Scaling such models requires sustained financing, workforce development, and adaptation to local health system constraints.

This study contributes by providing a structured analytical framework that explicitly links COVID-19 lessons to NiV preparedness gaps and translates these into actionable, context-sensitive policy priorities. By integrating cross-sectoral evidence and real-world outbreak experience, the review moves beyond descriptive synthesis to offer a coherent and scalable roadmap for strengthening global pandemic preparedness.

## 10 Conclusions

The inevitability of future pandemics is no longer a matter of speculation but a predictable outcome of ongoing ecological, societal, and virological dynamics. The COVID-19 crisis exposed both vulnerabilities and latent capacities within global health systems, underscoring that preparedness is not an accidental byproduct, but a deliberate policy choice shaped by governance, investment, and collective will. The emergence of NiV, a pathogen with high mortality, zoonotic origins, and pandemic potential, serves as a stark reminder that the next global health emergency may arise from familiar sources, yet presents novel challenges.

Lessons from COVID-19 have underscored the importance of robust surveillance at human–animal–environment interfaces, equitable access to diagnostics and vaccines, and the integration of One Health principle into public health strategies. However, persistent gaps in risk assessment, inter-pandemic investment in core capacities, and equity-driven response frameworks continue to threaten preparedness. Recent experiences demonstrate that effective pandemic readiness requires proactive planning, sustained scientific momentum, international collaboration, and political commitment to translate evidence into action.

In summary, although the precise timing or nature of the next pandemic cannot be determined, its occurrence is certain. The world stands at a crossroads: either heed the hard-won lessons of COVID-19 to build resilient systems capable of confronting threats such as the Nipah virus, or risk repeating cycles of unpreparedness with devastating consequences. The urgency to act is paramount; morbidity and mortality can be mitigated only through intentional policy choices when the next pandemic threat emerges.

## CRediT authorship contribution statement

**Sharmake Gaiye Bashir:** Conceptualization. **Ilyas Abdullahi khalif:** Methodology. **Yusuf Abdullahi Hubow:** Investigation. **Ahmed Mohamed Omar:** Methodology. **Narura Omar Mohamed:** Conceptualization. **Ayan Abdullahi Mohammed:** Validation. **Hibo hassan Mohamed:** Project administration, Methodology. **Nour Ahmed Dahir:** Resources, Project administration. **Aniso Mohamed Abdi:** Methodology, Investigation. **Abas Nor Abdi:** Investigation. **Yakub Burhan Abdullahi:** Conceptualization. **Mohamed Sharif Abdi:** Investigation, Conceptualization. **Naima Ibrahim Ahmed:** Investigation, Conceptualization. **Yusuf Hared Abdi:** Resources. **Ahmed Abdinasir Abdulle:** Writing – review & editing, Supervision.

## Informed consent

Not applicable.

## Organ donation

Not applicable.

## Ethical statement

Not applicable.

## Data availability statement

Data sharing is not applicable to this article as no datasets were generated or analyzed.

## Animal treatment

Not applicable.

## Generative AI

Not applicable.

## Funding

This research did not receive any specific grant from funding agencies in the public, commercial, or not-for-profit sectors.

## Declaration of competing interest

Authors declare that there is no conflict of interest.
